# Guidelines for wrist-worn consumer wearable assessment of heart rate in biobehavioral research

**DOI:** 10.1038/s41746-020-0297-4

**Published:** 2020-06-26

**Authors:** Benjamin W. Nelson, Carissa A. Low, Nicholas Jacobson, Patricia Areán, John Torous, Nicholas B. Allen

**Affiliations:** 1 Department of Psychology, University of Oregon, Eugene, OR USA; 2 Department of Psychiatry and Behavioral Sciences and Department of Rehabilitation Medicine, University of Washington, Seattle, WA USA; 3 Department of Medicine, University of Pittsburgh, Pittsburgh, PA USA; 4 Geisel School of Medicine, Dartmouth College, Hanover, NH USA; 5 Center for Technology and Behavioral Health, Dartmouth College, Hanover, NH USA; 6 Department of Psychiatry, University of Washington, Seattle, WA USA; 7 Beth Israel Deaconess Medical Center, Department of Psychiatry, Harvard Medical School, Boston, MA USA

**Keywords:** Psychology, Cardiovascular diseases, Risk factors, Biomarkers

## Abstract

Researchers have increasingly begun to use consumer wearables or wrist-worn smartwatches and fitness monitors for measurement of cardiovascular psychophysiological processes related to mental and physical health outcomes. These devices have strong appeal because they allow for continuous, scalable, unobtrusive, and ecologically valid data collection of cardiac activity in “big data” studies. However, replicability and reproducibility may be hampered moving forward due to the lack of standardization of data collection and processing procedures, and inconsistent reporting of technological factors (e.g., device type, firmware versions, and sampling rate), biobehavioral variables (e.g., body mass index, wrist dominance and circumference), and participant demographic characteristics, such as skin tone, that may influence heart rate measurement. These limitations introduce unnecessary noise into measurement, which can cloud interpretation and generalizability of findings. This paper provides a brief overview of research using commercial wearable devices to measure heart rate, reviews literature on device accuracy, and outlines the challenges that non-standardized reporting pose for the field. We also discuss study design, technological, biobehavioral, and demographic factors that can impact the accuracy of the passive sensing of heart rate measurements, and provide guidelines and corresponding checklist handouts for future study data collection and design, data cleaning and processing, analysis, and reporting that may help ameliorate some of these barriers and inconsistencies in the literature.

## Heart rate research in psychology and medicine

Heart rate (HR) has long been used as a clinical indicator of overall cardiac health. HR is predominantly influenced by the coordination of the sympathetic and parasympathetic branches of the autonomic nervous system, which can be modified by biopsychosocial factors including physical and relational stress, diet, physical fitness, medication use, and substance use^1^; thus, offering a direct and quantifiable connection to the stress diathesis model of health. In this model, cardiovascular dysfunction has been proposed to be a putative mechanism associated not only with morbidity and mortality, but also with a range of psychiatric disorders.

In terms of physical health, meta-analyses have shown that resting HR above 80 beats per minute (bpm) is associated with a 33% increased risk for cardiovascular mortality and a 45% higher risk for all-cause mortality^2,3^. In terms of HR dynamics, delayed HR recovery after a stressor (i.e., the return to resting or baseline HR) has also been shown to be associated with mortality^4^, whereas the literature is more mixed on the association between HR reactivity (i.e., the increase in HR from resting or baseline due to psychobiological stressors) and health outcomes^5^. Furthermore, dysfunction of the autonomic nervous system is also associated with a range of psychiatric disorders^6,7^ including anxiety disorders^8^, depressive disorders^9^, posttraumatic stress disorder^10^, and schizophrenia^11^. In a large prospective, Swedish study of over 1 million male participants, elevated resting HR during late adolescence was found to be associated with an increased risk of obsessive-compulsive disorder, anxiety diagnosis, and schizophrenia, whereas lower resting HR was associated with substance use disorders and violent criminality^12^.

In addition to these studies, there is strong evidence that psychiatric diagnoses are associated with increased risk for physical morbidity and mortality. For example, those with psychopathology lose a median of 10 years of life^13^, with the most common cause of death due to cardiovascular diseases^6^. Cardiac activity therefore may be a biological factor that serves as a mechanism linking mood and anxiety disorders with cardiovascular and metabolic risk and mortality^14,15^. In other words, HR may be a transdiagnostic biomarker with clinical utility for psychology, psychiatry, and medicine to improve nosology and increase identification of those at risk for the onset of psychiatric and physical health disorders. Until recently, the quantification and measurement of HR has been relegated to medical and research settings, which has largely precluded the observation of HR during patient and participant daily life, thereby limiting the generalizability of findings to real-world living conditions.

## Current limitations in cardiovascular research

The foundation of HR research has, until recently, been largely constrained due to (1) lack of ecological validity (i.e., the degree to which a laboratory or medical environment represents actual real-world conditions), (2) inability to collect temporally detailed and longitudinal HR measurements both within and across time (e.g., hours, days, weeks, months), and (3) prohibitive financial cost of devices for everyday use.

In terms of ecological validity, HR recordings have historically been collected in discrete laboratory or medical environments that do not represent daily life and as such, can be anxiety-inducing as medical phobia is common and novel environments may increase HR, which consequently could lead to overestimation of resting HR^16^ as reflected in greater adrenergic activity in clinical settings - a known phenomenon called “white-coat hypertension or syndrome”^17,18^. In other words, research has been historically limited to collecting data from medical patients and research participants in novel settings that might not generalize to their everyday lives, which creates the potential for inaccurate baselines and therefore inaccurate measurement of HR dynamics (i.e., reactivity/recovery). The law of initial values gives further insight into this issue by highlighting that an initial value has a large impact on the strength and direction of a response, such that a higher pre-stimulus level will lead to a smaller post-stimulus response or, in other words, a smaller response when there is a greater initial value^19–21^. This is similar to a ceiling effect limiting the magnitude of a reactivity response and this can artificially constrain the range of measurements (e.g., reactivity) within a specific construct, such as HR.

Second, collection of HR data in these environments lacks temporal resolution within and across days as HR is usually collected during a short recording within a single day, which has precluded research from comprehensively investigating the transition from acute to chronic stress^22^. Greater temporally detailed longitudinal HR data collection may reveal important within day as well as weekly, monthly, seasonal, and yearly fluctuations in HR that are currently not captured and may contain important diagnostic and predictive information. Lastly, the accurate collection of HR has historically been financially restrictive relegating these measurements to medical and scientific contexts, and precluding the large-scale adoption and democratization of these technologies by the general public.

## Wearable technological advancements and the scalability of cardiovascular research

Currently, wrist-worn wearables provide an opportunity to examine HR in real-world environments, across longer periods of time, and with high resolution at a low cost, by using an optical sensor (photoplethysmography; PPG), that allows these devices to collect volumetric changes in blood profusion (i.e., pulse rate) that can serve as a surrogate for HR (see Table 1 for summary of most popular consumer wearable devices). Wearable PPG sensors can use different colors of light, such as green or red, to index these changes in blood profusion with each having different costs and benefits. For example, green LED light is a shorter wave-length and has been shown to be convenient, because it has good signal-to-noise ratio and is resistant to motion artifacts, but is limited by the amount of light that can pass through tissue, especially darker skin tones. In contrast, red LEDs, which are more commonly used in medical settings, are not absorbed as well by the skin allowing the light to transmit deeper and allowing for detection of multiple biological measures, but it has a lower signal-to-noise ratio and is more susceptible to motion artifacts^23^. Overall, PPG devices allow for in vivo examinations of the roles that resting HR and HR dynamics (e.g., reactivity and recovery) have in medical and psychiatric disorders in real-world settings. It is important to note that while HR and pulse rate are often used interchangeably, they are two distinct physiological signals with HR representing the heart contraction via electrical impulses, whereas pulse rate represents the rate of change in blood pressure due to the ventricular ejection of blood. The latter of these two is the predominant method for data collected with wrist-worn wearable devices, although some more recent wrist-worn wearable devices, such as the Apple Watch Series 4 and 5, have allowed for the collection of HR via electrical impulses on par with a single-lead electrode. Currently, this technology is limited, as users have to sit still with their watch wearing wrist resting on a flat surface and then close the circuit by putting a finger from the hand opposite to the watch for 30 s. Therefore, this novel electrocardiogram (ECG) technology only allows for the discrete, rather than continuous, collection of data, which precludes the ability for temporally detailed longitudinal ECG collection at this time. For this reason, addressing accuracy of wearable ECG sensors is outside the scope of the current review and will not be discussed further. Furthermore, PPG can be used to index heart rate variability (HRV), blood pressure, oxygen saturation, and cardiac output^24^, but as of yet have not be widely rolled out in consumer-use devices and therefore data collection other than HR will not be discussed further.

**Table 1:** Summary of most popular consumer wearable devices.

Device^a^	Sensor type				FDA status	Sampling rate (i.e., how often it samples)	Sampling frequency	Cost	Market size/share 2019 Q4^78^
	Green LED	Red LED	Infrared LED	ECG					
Apple^79^	X		X	X	510(k) class II clearance^b^	Variable • Rest—every 10 min • Exercise—continuous	100 s × per second	$199 (v3) to $399 (v5)	36.5%
Fitbit^80^	X				None	Variable • Rest—5 s intervals • Exercise—1 s intervals	Unknown	$149 (Charge 4) to $199 (Versa 2)	5.0%
Garmin^81^	X				None	Variable	Variable • High Frequency • Low Frequency	$129 (vivosmart 4)	^a^
Samsung	X				None	Manual	Unknown	Galaxy Fit ($99)	8.8%
Xiaomi	X				None	Continuous	Unknown	Mi Band 4 ($39.99)	10.8%
Huawei^82^	X				None	Default is set to manual. Can turn on continuous, which measures every 10 min or every 6–10 s during high-intensity workouts	Unknown	Huawei Band 4 ($42.31)	7.8%

ECG, electrocardiogram; LED, light-emitting diode.

^a^Data are presented on most recent wearable devices for each manufacturer. Garmin was not included in the top 5, so was listed under other.

^b^This only applies to the ECG sensor and high heart rate notifications.

## Increased popularity of wrist-worn wearable devices

Researchers are increasingly using consumer wearables or wrist-worn smartwatches and fitness monitors for the digital measurement of cardiovascular health and psychophysiological stress as these devices allow for continuous, scalable, and unobtrusive, “big data” collection of overall cardiac activity in real-world conditions with large samples^25–30^. Between 2008 and 2018 there was a 580% increase in total articles published that contained “wearable” and “heart rate”, indicating that this is a burgeoning area of research that may benefit from standardization of procedures to increase generalizability and clinical utility of research findings.

Recent information on the reproducibility and replicability crisis has swept the sciences and as such these issues are very important for digital health and mHealth research, which includes wrist-worn HR accuracy and any studies that utilize wearable HR data, as this new field will allow for large data samples^31^ with various sensors that will increase the ease for p-hacking. Recent research has proposed using wearables in psychological research^32^ and treatment settings^33^, whereas other research has already begun to use mHealth data to show important global real-world HR norms^16^, global differences in HRV by age, gender, and time of day^26^, and allowing for surveillance of influenza symptoms^34^, yet questions remain as to the accuracy and validity of data collected from wearables^35,36^.

## How accurate is HR detection with wrist-worn wearable devices?

Current research into the HR accuracy of wrist-worn wearable devices attempt to compare the measurement between wearable PPG and a reference method, such as an ECG, in order to determine whether or not measurements between the devices are outside of clinically important limits of agreement, so that devices can supplement, eventually replace, or be used interchangeably^37,38^. Although some studies have attempted to address wearable HR accuracy using the gold-standard reference method of ECG, which directly measures HR via electrical impulses from the heart and is used for clinical accuracy^38–44^, other studies have used suboptimal reference methods, such as the chest straps^45–47^ or pulse oximeters^48^, which themselves have a degree of error (varying between 0 and 40 bpm when compared to ECGs^44^). This has the potential to introduce a degree of additional error into wrist-worn wearable HR accuracy research as suboptimal referencing methods can introduce unnecessary measurement error and potentially undermine findings. Despite this caveat, current research into wearable HR accuracy is promising as is discussed below. Furthermore, some wearable devices, such as the Apple Watch 4 and 5, have gained FDA 510(k) class II clearance for the ECG feature and ability to detect arrhythmias^49^, indicating that FDA clearance and conformance with IEC-60601(−2–47) guidelines may provide a path towards standardization of feature sets and firmware for consumer wearables in the future, which raises the important question of the interface between consumer wearables and medical devices. Currently, in most instances, consumer wearables are not medical devices, but this is an issue that is evolving and in the future may influence legislation and standardization. Below we mainly focus on HR accuracy of Apple Watch and Fitbit as these are two of the most popular wearable companies in the United States and have been more commonly examined in empirical research to date, but it should be noted that other consumer wearable brands, such as Garmin^45,46,50^, Samsung^42,43,48,51^, and Microsoft^42,47^ have also had studies examining their HR accuracy.

Current research verifying wearables to the gold-standard ECG, which uses electrodes to directly measure cardiac muscular contractions from electrical activity of the heart, have shown that on average wearables slightly and negligibly underestimate (−0.9 to −7.2 bpm) absolute HR^40,41,44,52^ with the Apple Watch having slightly greater accuracy than Fitbit devices (Apple Watch: −1.3 bpm, Fitbit: −9.3 bpm^42–44^), as well as lower overall error with the Apple Watch models ranging from 1.20 to 6.70% error and Fitbit models ranging from 2.38 to 21.36% error^39,42,45^, lower mean difference standard deviation^43^, and higher agreement with ECG than Fitbit devices^39,44^. Furthermore, recent research has indicated that consumer wearables, such as the Apple Watch and Fitbit are more accurate than research-grade wearables, which are much more expensive yet provide the benefit of raw data^50^. These findings have led some researchers to conclude that wearables detect HR with acceptable accuracy in both laboratory and real-world settings for research, but importantly not medical settings^38,42^ as more research needs to be done. For example, researchers compared the accuracy of ambulatory ECG, Apple Watch 3, and Fitbit Charge 2 data within an individual across 24 h and found that while devices slightly underestimated HR when values were aggregated within and across different daily activities, which indicated high HR accuracy, any individual HR observation could deviate from ECG by significantly large margins that would be likely be problematic in medical settings^38^. This finding indicated that while overall, summary statistics after outlier detection and data cleaning may be very accurate for research purposes, any single observation in real-time may have a large degree of error, which could be significant for moment-to-moment observations in medical settings. These preliminary findings suggest that researchers can utilize wearable HR to accurately assess HR, especially resting HR, when using aggregated data across activities and removing outliers. The next steps for consumer wearable HR device accuracy research will require meta-analyses in order to summarize the current state of consumer wearable HR accuracy. Despite the promise of wearable HR accuracy, replicability and reproducibility in the future utilizing wearable HR as a transdiagnostic marker of psychiatric and physical health will likely be limited due to lack of standardization of data collection, study design, and data processing as well as inconsistencies in the reporting of technological, biobehavioral, and demographic characteristics that introduce unnecessary noise into the literature and cloud interpretation and generalizability of findings.

## Disadvantages to non-standard reporting

The lack of “metadata standards” or guidelines and standards for describing, reporting, and creating meaning from collected data in order to increase transparency, accountability, and interpretability of data in mHealth is likely to create a number of issues related to noise/signal ratio, contradictory results, lack of generalizability, and false positive and negative findings, which have historically undermined replicability (i.e., the ability to replicate a study with a new data set) and reproducibility (i.e., the ability to replicate findings from the same data set). Therefore, many researchers have begun to push for increased focus on reproducible and open practices in science^31,53–55^. Below we outline data collection, cleaning, and protocol design issues as well as technological, biobehavioral, and demographic factors that may influence reproducibility and replicability before turning to guidelines that the field can use in order to increase scientific rigor and generalizability of findings.

## Study protocol design and data collection: addressing design, biobehavioral, and demographic issues that can influence reproducibility and replicability

There are many researcher degrees of freedom when it comes to decisions related to study protocol design and data collection, cleaning, and processing that are important to note as standardizing this process may help ameliorate some barriers and inconsistencies in the literature.

### Protocol design

#### Summary metric calculation

Hardware differences between devices determine HR measurement second to second, which has large implications on overall interpretability of the data, such that sampling rate can introduces researcher degrees of freedom for calculating bpm. Unfortunately, there is currently a lack of transparency from manufacturers on underlying algorithms and raw data outputs are often times not provided, which prevents researchers from dealing with this issue and creating a standardized protocol for transforming raw data to HR. For example, Fitbit logs HR data at 1 s intervals during exercise tracking and 5 s intervals at other times, whereas the Apple Watch samples HR data every 10 min, except during workouts, which are continuous. Relatedly, some devices, such as the Apple Watch can take multiple HR measurements within a minute allowing researchers to use various calculations, such as the mean or median within this minute to calculate bpm, which may alter wearable data values. Researchers should report how bpm or other summary metrics are calculated (e.g., mean or median). Therefore, summary metric calculation should also be clearly reported in publications.

#### Data cleaning: outlier detection and dealing with missing data

In addition, the methods researchers select to clean and process raw data prior to analyses can also affect results and conclusions and should be reported in detail. These choices include how missing data values are handled (listwise deletion, pairwise deletion, and multiple imputation), and how outliers or biologically implausible values are handled (e.g., some devices record HR values of zero when devices are not worn).

Data cleaning can be an important step in data pre-processing in ambulatory HR assessments. There is a strong need to customize the methods of data cleaning to the device being used and the models being adopted, rather than adopting a universal pre-processing pipeline due to device-specific differences and based on the study research questions. For example, outliers in linear models may strongly impact linear estimators, and some studies may therefore adopt a modeling strategy wherein values are winsorized (e.g., extreme values can be truncated at the 2.5th and 97.5th quantiles^56^). However, if a study were interested in HR arrythmias using wearable assessments (such as atrial fibrillation), then these extreme values may be of vital interest and should not be removed or altered^27^. Moreover, for some, but not all devices will report missing HR data with zero values and these values should be removed and filled in with missing data rather than winsorized (which would otherwise substantially bias parameter estimates unless the degree of missing HR points was trivial). Other strategies (e.g. spectral frequency filters), will depend upon the type of the sensor being utilized in the wearable sensor^57^. Rather than proposing universal standards, we rather recommend that researchers thoughtfully approach data cleaning, altering their decisions to both their own research questions and to the devices being used, and reporting their utilized methodology.

The practice of completely removing persons from studies due to proportions of missing data (i.e. listwise deletion) is known to bias estimation and adversely impact the standard error of point estimates across modeling strategies^58^. More specifically, simulation studies with intensive longitudinal data suggest that multiple imputation and full-information maximum likelihood reliably produce better parameter estimates than listwise deletion^59^. Moreover, dynamic models of HR captured in daily life can be effectively estimated even with very large proportions of missing data (e.g. >70% missingness for each subject) using full-information maximum likelihood estimation^60^. Based on this, we recommend that authors aim to be as inclusive as possible, using appropriate model-based strategies for dealing with missing data.

#### Defining non-wear-time: device charging and non-adherence

For long-term field studies, information about participant adherence and wear-time should be considered and reported since these issues can affect data quality. The definition of wear-time and any minimum wear-time thresholds for data inclusion (e.g., days with wear-time less than 1000 min excluded^34^; valid day of wear-time defined as at least 600 1-min epochs of nonzero HR values within a calendar day)^61^. One issue reported by multiple users on the online Fitbit Community Forum is occasional logging of HR values (often higher and more variable than typical, e.g., 100–150 bpm) when the devices are not being worn, suggesting that supplemental non-wear-time diaries may be required for some devices or protocols.

#### Standardizing wrist data feature collection

There are a number of study design features related to the wrist that have been shown to have effects on HR accuracy. Larger wrist circumference has been shown to be associated with reduced HR accuracy^42^, although these outcomes have not been found in all studies^44,46^. In addition to wrist circumference, there are a number of other factors including wrist placement, tightness of device, dominant vs non-dominant hand use, and degree of wrist movement that have consistently been shown to influence the accuracy of HR measurement^39^, which may be due to the fact that greater wearable movement is associated with decreased PPG accuracy as discussed below^62^. These are likely some of the most important factors influencing HR accuracy. As such, wrist placement, tightness of device, dominant vs non-dominant hand use, and whether wearable devices are naturalistically placed by participants as opposed to explicit instructions by experimenters should be reported.

#### Open science and data transparency

Lastly, from a study design perspective, mHealth research will be particularly susceptible to a number of research practices, which have been shown to degrade the quality, consistency, generalizability, and therefore replicability and reproducibility of findings^31^ as data sensors in this research field allow for the vast collection of big data samples, which will be vulnerable to some of the “four horsemen of the reproducibility/replicability apocalypse”, including (1) publication bias^63^, (2) P-hacking, (3) hypothesizing after the results are known or HARKing^54^, and (4) low power^64^, the last of which likely will not be an issue for mHealth research as large samples sizes will be relatively easy to collect even in samples with low base rates in the population. Therefore, we urge future wearable HR studies and digital passive sensing mHealth studies in general to take up free open science practices (http://osf.io/), such as study preregistration, open code, and open data and where open data cannot be released due to clinical population or privacy issues, releasing simulated data sets that preserve data set characteristics where applicable^65^. On the editorial and reviewer side, we join other researchers in urging for editors and reviewers to allow for null, inconclusive, and contradictory data results without putting undue pressure on researchers to come up with coherent narratives that might not fit the data^66^.

### Biobehavioral and demographic data

Individual differences and variability in both biobehavioral and demographic factors have the potential to influence the collection of HR during research studies and should therefore be strongly considered in descriptive reporting as well as statistical modeling in studies using HR measurement collected from wearable devices.

#### BMI and biological sex

Higher body mass index, which likely covaries with larger wrist circumference as described above, has been shown to be associated with reduced HR accuracy^42,62^, although again these outcomes have not been found in all studies^44,46^. Nevertheless this variable should be collected, reported, and potentially controlled for in research. In addition, research has not always reported participant biological sex, but research has found higher device error in males compared to females^42^, indicating that participant biological sex should be collected and possibly controlled.

#### Skin tone and hair follicle density

Evidence is starting to accumulate on the importance of skin tone when it comes to accuracy and generalizability of wrist-worn wearable studies with darker skin tone (and tattoos) being found to be associated with reduced wrist-worn HR accuracy as they absorb more green light^67^, yet this is not found in all studies^43,50^. The importance of this point cannot be overstated as historically, science and medicine have paid less attention to recruiting minority groups^68^, which has resulted in blunted health gains in these populations making it of utmost importance to identify how HR accuracy varies by race and ethnicity factors in order to ensure that findings generalize to these populations as future research might have direct implication for patient healthcare. Currently, the vast majority of wrist-worn wearable studies incorporating HR measurements do not report or control for participant race and ethnicity, which undermines the ability to look at potentially important group differences in health outcomes and therefore decreases the ability of results to generalize to large segments of the population. These demographic characteristics should be collected, reported, and controlled for in all wearable HR studies moving forward and researchers should collect data related to participant skin tone (e.g., Fitzpatrick skin type). Furthermore, wearables that use red light will likely be more effective on darker skin tones as it is not absorbed by melanin, which may allow for more accurate HR measurements^23^. In addition to skin tone, some research indicates that hair follicle density and sweat can influence device measurement^69^.

#### Motion artifacts and physical activity

Importantly, two related factors identified as influencing wearable HR accuracy are motion artifacts^23^ and level of physical activity^46^. Specifically, research has shown that at rest, wearables can perform similarly to an ECG, with some research showing that absolute error during activity is 30% higher than rest^50^, and a substantial amount of research showing that wearable devices are more accurate during rest and low intensity exercise when compared to higher intensity exercise, when wearable devices can begin to deviate more from ECG recordings^38,39,44,46,70–73^, although this has not been found in all studies^42,45,47,51^, with some studies finding that accuracy of HR measurement was comparable across resting baseline or vigorous activity^45^, whereas a second study found that the accuracy of HR measurement was highest during running^51^, and a third found that measurement during walking, running, and cycling was more accurate for some devices than during sitting^42^. Other research found that across four consumer wearables and two research-grade devices all were “reasonably accurate” during rest or during prolonged HR that was elevated and that differences in accuracy tended to arise during changes in activity^50^. Therefore, it is possible that activity intensity may be less important to device accuracy than the degree of erratic wrist movements and corresponding position of the wearable device during an activity^74^, which cause motion artifacts and may or may not co-occur with more vigorous physical activity. Furthermore, research has described the “cross-over effect” when repetitive motion and activity causes underlying algorithms to mistake the movement cadence for cardiac activity^50,75^. As mentioned previously, one strength of PPG green light is that it is less susceptible to motion artifacts, whereas PPG red light is more susceptible to motion artifacts indicating that wearables that use green PPG or a combination of green and red PPG are likely to perform better during movement and physical activity^23^. Future studies may want to control for wearable actigraphy data in order to remove HR accuracy variance that is influenced by wrist movement.

### Technological factors that can influence reproducibility and replicability

In addition to the high heterogeneity in study protocol design as described above, various technological factors have the potential to influence the collection of HR during research studies and should therefore be strongly considered in descriptive as well as statistical modeling when using wearable devices. Specifically, due to the variety of wearable devices within and across technology companies, versions of devices, and the fast pace of technological advancement (especially in consumer products) there are a number of technological factors that should be reported and potentially controlled for in mHealth studies. First, as described above, there are currently various wearable devices and versions on the market that have the potential to use different hardware, which may introduce differences in HR recordings. One such hardware difference between devices is sensor type. For example, the Apple Watch uses both PPG green light-emitting diode (LED) lights and infrared LED light, whereas Fitbit uses only PPG green LED.

In addition to hardware differences, researchers should also consider software differences between devices. Wearable manufacturers use proprietary algorithms to translate PPG signals to HR measurements, which may influence accuracy^23^. These algorithms, while often simplistic, may be altered with firmware updates, yet most studies fail to report firmware information. Not controlling for firmware version may lead to poorer reproducibility and replicability as two studies investigating the same device with two or more different firmware versions might actually come to different conclusions even if all other variables are held constant.

Due to the high heterogeneity in device type, version, hardware, software, and sampling rate, these should be consistently collected and reported in descriptive tables for both cross-sectional and longitudinal studies. In addition, if a study uses multiple types of devices or a single device in a longitudinal study that covers a period of time when there are firmware updates, then statistical models should control for device type and firmware version.

## Guidelines

Here we join other researchers in the call for the establishment of “metadata standards” or guidelines for collecting, processing, describing, reporting, and creating meaning of collected HR and other biological data in order to increase transparency, accountability, and interpretability of data^28,76,77^. With this in mind, we have created two checklists. The first checklist (Table 2) addresses standards for reporting study characteristics, including how the study was designed, technological hardware and software used; participant characteristics; and data cleaning, analysis, reporting, and transparency. Note that we suggest that researchers report the reliability of their metric and justify why this reliability threshold is sufficient for the given study. We make this recommendation purposefully and do not adopt a single global threshold for all of research. We do this because research is heavily context dependent. For example, a very high standard might be needed when HR is used to classify heart disease. However, in contrast to this situation, when HR is only one of many potential avenues of information it might be sufficient that HR signal is reliably better than complete noise. For instance, adopting a merely better-than-noise standard in a multi-component machine learning model could still improve performance and may be preferable when researchers are focused on scalable low-cost devices. In the second scenario, imposing an arbitrary standard might make a model less accurate because it might otherwise require the researchers to drop HR signal entirely.

**Table 2:** mHealth wearable heart rate metadata checklist 1: descriptive reporting of sample.

Describe	
Study design protocol	
Naturalistic or laboratory	□
Group (Psychiatric group diagnostic or symptom selection criteria)	□
Recruitment source	□
Inclusion/exclusion criteria (presence or absence of conditions, age range)	□
Technological factors	
Device manufacturer	□
Device type	□
Device version	□
Firmware version	□
Hardware (sensor type)	□
Sampling rate	□
Device reliability and justification for why this level of reliability is adequate for the study design	□
Participant characteristics	
Age	□
Race	□
Ethnicity	□
Biological sex	□
Gender	□
Skin tone (e.g., Fitzpatrick skin type)	□
Body mass index	□
Wrist circumference	□
Wrist placement (e.g., dominant or non-dominant)	□
Medical condition	□
Cardioactive medication use	□
Data cleaning, handling, and analysis	
Summary bpm metric calculation (e.g., mean, median)	□
For multiple samples per minute, how was final bpm calculated	□
Definition of non-wear-time and reason for data loss (battery life, device failure, participant attrition)	□
Dealing with missing data	□
Listwise deletion (not recommended)	□
Pairwise deletion (not recommended)	□
Model-based (e.g. full-information maximum likelihood, multiple imputation)	□
Outlier identification and correction	□
Wrist circumferenc	□
Wrist placement	□
Dominant or non-dominant	□
Tight vs loose	□
Naturalistic use by participants vs explicit instructions by experimenters	□
Data reporting	
Reporting descriptives (e.g., mean, standard deviation, range for overall sample and by group)	□
Data transparency	
Preregistration	□
Large passive sensing data sets will be ripe for p-hacking. Pre-register analyses prior to viewing data collected or try to draw predictions from out of sample predictions.	□
Open code and data	□
Provide access to code and data, if applicable	□

The second checklist (Table 3) provides potential covariates in regards to technological factors and both biobehavioral and demographic factors that have also been shown to influence accuracy of HR measurements and dynamics. We propose that future research utilizing wearable HR should report in methods sections that study design protocol, biobehavioral, participant demographic, and technological variables have been reported in accordance with these standardized guidelines and include these checklists in supplemental materials. Utilizing these guidelines and referencing them in methods sections has the potential to decrease non-standard reporting, increase reproducibility and replicability, while also leading to greater generalizability of results, which may increase the clinical utility of findings in this field.

**Table 3:** mHealth wearable heart rate metadata checklist 2: potential covariates.

Control	
Technological factors	
Device manufacturer	□
Device type	□
Device version	□
Firmware version	□
Hardware (sensor type)	□
Sampling rate	□
Device reliability and justification for why this level of reliability is adequate for the study design	□
Participant characteristics	
Age	□
Race	□
Ethnicity	□
Biological sex	□
Gender	□
Skin tone (e.g., Fitzpatrick skin type) (e.g., Fitzpatrick scale)	□
Body mass index	□
Wrist circumference	□
Wrist placement	□
Dominant or non-dominant	□
Tight vs loose	□
Naturalistic use by participants vs explicit instructions by experimenters	□
Medical condition	□
Cardioactive medication use	□

## Conclusion

HR has long been used as a clinical indicator of overall cardiac health that has been conceptualized as a transdiagnostic biomarker with clinical utility in psychology, psychiatry, and medicine to improve nosology, and increase identification of those at risk for the onset and relapse of psychiatric and physical health conditions. Cardiovascular dysfunction has been proposed to be a putative mechanism associated not only with morbidity and mortality, but also with a range of psychiatric disorders. Until recently, real-world continuous collection of HR in medical patient and research participant lives has been limited by technological and financial factors. The recent introduction of consumer wearables or wrist-worn smartwatches and fitness monitors for the digital measurement of cardiovascular health and psychophysiological stress has allowed for the continuous, scalable, and unobtrusive, “big data” collection of overall cardiac activity in real-world conditions with large samples that allow for the collection of passive, large-scale, and ecologically valid data. This has the potential to improve the study of cardiovascular health in medicine and psychophysiological stress in psychiatry and psychology.

The current literature indicates that these devices can provide acceptable accuracy for the measurement of HR for research settings, especially when scalability and ecological validity of measurement settings are critical considerations that may outweigh the need to offer gold-standard assessment. It is important to note that PPG technology can be used to index blood pressure, oxygen saturation, and cardiac output, which will likely be slowly rolled out into wearable technology moving forward. In addition, PPG can index HRV, which is derived from beat to beat intervals calculations in the time-domain, such as root mean squared of successive differences or the standard deviation of NN intervals, the latter of which is used in the Apple Watch, but only with sporadic recordings throughout the day. Currently, many commercial wearables do not provide beat to beat interval data to users, although some researchers have worked with industry partners to derive measurements of HRV^26^. The guidelines provided above will be necessary to apply to research when studying PPG data at more fine grained detail than HR.

Given the variations in both hardware and software in these devices, variation in study design protocol, as well the way in which individual differences across both biobehavioral and demographic characteristics may affect the accuracy of measurement, the field requires standards of reporting that will at the very least allow for the characterization of these factors when comparing studies. In this paper, we have proposed a pair of reporting checklists that indicate the details that should be included in any paper using this form of HR measurement.

Overall, although consumer wearable devices that collect HR likely are not yet advanced enough to be replacing medical-grade ECG anytime soon, these devices can be used to supplement medical-grade devices and continuously collect HR data on research subjects when risk for acute cardiac events is not of immediate concern. This has the potential to advance research into cardiovascular health and psychophysiological stress in order to better understand how HR influences overall physical and psychological health.
